# Building general practice training capacity in rural and remote Australia with underserved primary care services: a qualitative investigation

**DOI:** 10.1186/s12913-019-4078-1

**Published:** 2019-05-28

**Authors:** Louise Young, Raquel Peel, Belinda O’Sullivan, Carole Reeve

**Affiliations:** 1 College of Medicine and Dentistry, James Cook University, 1 James Cook Drive, Townsville, QLD 4811 Australia; 2 Monash University School of Rural Health, Bendigo, Victoria Australia

**Keywords:** General practice training, Family Physician Training, Primary Care Services, Rural Health, Remote Underserved Communities, Medical Workforce Shortage, Health Care Equity, Qualitative Research, Thematic Analysis

## Abstract

**Background:**

Australians living in rural and remote areas have access to considerably fewer doctors compared with populations in major cities. Despite plentiful, descriptive data about what attracts and retains doctors to rural practice, more evidence is needed which informs actions to address these issues, particularly in remote areas. This study aimed to explore the factors influencing General Practitioners (GPs), primary care doctors, and those training to become GPs (registrars) to work and train in remote underserved towns to inform the building of primary care training capacity in areas needing more primary care services (and GP training opportunities) to support their population’s health needs.

**Methods:**

A qualitative approach was adopted involving a series of 39 semi-structured interviews of a purposeful sample of 14 registrars, 12 supervisors, and 13 practice managers. Fifteen Australian Medical Graduates (AMG) and eleven International Medical Graduates (IMG), who did their basic medical training in another country, were among the interviewees. Data underwent thematic analysis.

**Results:**

Four main themes were identified including 1) supervised learning in underserved communities, 2) impact of working in small, remote contexts, 3) work-life balance, and 4) fostering sustainable remote practice. Overall, the findings suggested that remote GP training provides extensive and safe registrar learning opportunities and supervision is generally of high quality. Supervisors also expressed a desire for more upskilling and professional development to support their retention in the community as they reach mid-career. Registrars enjoyed the challenge of remote medical practice with opportunities to work at the top of their scope of practice with excellent clinical role models, and in a setting where they can make a difference. Remote underserved communities contribute to attracting and retaining their GP workforce by integrating registrars and supervisors into the local community and ensuring sustainable work-life practice models for their doctors.

**Conclusions:**

This study provides important new evidence to support development of high-quality GP training and supervision in remote contexts where there is a need for more GPs to provide primary care services for the population.

## Background

Approximately 29% percent of the Australian population lives in rural and remote areas [1] where their health status is worse than those living in major centres, with higher mortality rates for chronic disease, injury and poorer access to and use of health services [2]. Australians living in rural and remote areas have access to 274 doctors per 100,000 in remote/very remote areas compared with 433 doctors per 100,000 in major cities [3]. The health sector employs more people than any other industry in Australia, but the maldistribution of health workers continues to be problematic and undermines the capacity to achieve health improvements for rural and remote people whose cultures, lives, and livelihoods are based in these towns [4, 5].

Access to primary care services is particularly concerning for ensuring early intervention, continuity of care, and managing important health needs. However, in terms of more nuanced targeting of primary care workforce development, it is critical to identify communities where primary care training and primary care services need to be built up to adequately address population health needs. This includes understanding how to increase General Practice (GP), or family physician training posts in these specific communities. This paper describes the culmination of such work, undertaken in the context of north-western remote Queensland, Australia.

McGrail et al. [6] undertook a quantitative analysis in 2017 to explore the distribution of GP supervisors and registrars (doctors training in non-hospital community-based training posts to become GPs) relative to general practice (GP) workforce supply measures (GP billing data) and population needs according to defined rural and remote sub-regions and towns of north-western Queensland. Multiple standardised workforce indicators: supply, rurality, and other indicators, including population size, Australian Standard Geographical Classification – Remoteness Areas (ASGCRA; [7]), Modified Monash Model (MMM; [8]), Registrar count [6], aggregated Districts of Workforce Shortage (DWS) ratings [8–10], Index of Access (IA; [11–14]), Socio-Economic Indexes for Areas (SEIFA; [15]), and Indigenous population were applied to this evaluation.

A range of communities (*n* = 11), pre-identified from the McGrail et al. [6] study, were purposively sampled for the current study with the aim of more in-depth exploration of these contexts to understand the nature of work, supervision, and how to build general practice (GP) training capacity in these specific communities.

Building GP training capacity in these communities has enormous potential to provide additional primary health care services for populations in need of more services. GP registrars increase the available pool of local doctors seeing patients. Providing training in these settings is essential to develop GP registrars with relevant skills for the scope of practice required by remote communities. There is also the potential that registrars may stay in these communities after they complete their vocational training [16]. Rural-based GP training has been shown to increase the likelihood of GPs practising in these areas for at least five years [17]. Other research has shown that both rural background [18–20] and extended rural placements during medical school [21, 22] positively influence rural practice in early career. However, there is very little contextual information about the rich range of background factors related to working and undertaking supervised postgraduate medical training in such communities, making it hard to implement solutions.

This project was led by James Cook University’s (JCU) GP training program, Generalist Medical Training (GMT), which operates as one of nine decentralised regional GP training organisations across Australia. It provides GP training across over 90% of the large state of Queensland, much of it in rural and remote locations. In Australia, GP training can be commenced as early as the second year of postgraduate medical practice (after internship to achieve full registration). JCU’s goal is to provide training to build a distributed medical workforce with the skills to meet the population health needs of the large rural and remote catchment population. Hence, this project had high practical application to JCU and it was done in an academically rigorous way to inform the international evidence-based literature.

## Methods

### Participants

Eleven purposively selected towns were chosen for this study. They had been delineated as underserved for general practitioners as well as GP supervisor and registrars, relative to their assessed population need as per the McGrail et al. [6] study. These included towns with populations of < 15,000 people or were more than 10 km from the nearest regional centre with a population of 15,000 or more. Most had a district hospital (small rural hospital with selected generalist services) as a major referral site for surrounding communities and at least one general practice clinic in the town. Queensland remote areas has a higher than average proportion of Indigenous population and this was notable in several of the communities studied. The characteristics of the towns sampled are outlined in Table 1.

**Table 1:**
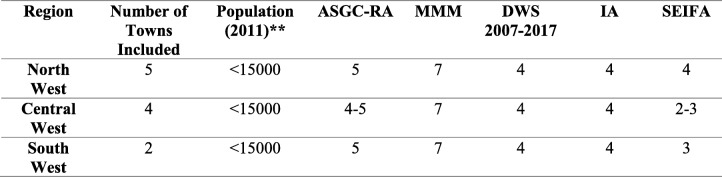
Selected Regions and Towns relative to population needs*

* Individual towns, registrar count, and indigenous population are not identified in this table for the purposes of adhering with ethical requirements and to keep towns and participants unidentified

** Population of 2011 as per the Australian Bureau of Statistics 2011 Census [23]

Within these towns all GP supervisors, GP registrars, and practice managers were invited to participate in a semi-structured interview face to face or remotely by phone, about supervising or receiving GP training in the context of their town. The basis for selecting these participants was to understand the factors from different perspectives as these can vary between GPs-in-training, longer term GPs, and those working in a business context. Interviews were conducted from mid-November 2017 to mid-February 2018, digitally recorded, transcribed verbatim, and entered into NVivo 11 Plus (QSR International Pty) for analysis. All transcripts were sent to participants for checking and confirmation prior to analysis.

### Data analysis

Qualitative data from semi-structured interviews underwent thematic analysis using a three-level qualitative approach [24]. Transcripts were read in full and coding of identified themes was confirmed using shared coding sessions and theme generation by two researchers (LY, RP) with consensus used to resolve discrepancies. Inter-coder reliability was undertaken by three researchers (LY, RP, BO’S) on half of a random selection of the transcripts to ensure consensus of themes and integrity of coding. Authors discussed and reached consensus about the final main themes and sub-themes (level 1 and level 2) as they emerged, further analysing and discussing the data over a period of six months. In addition to thematic coding, quantitisation as a mixed methods approach that allows the numerical translation, transformation or conversion of qualitative data, was applied to determine the importance and occurrence of each theme [25]. For this, qualitative verbal comments in sentence units were transferred into numerical form to show commonality of themes and to aid interpretation as to the weight of the data in each theme. Some verbatim illustrative quotes were selected from these sentence units and were included in the textual presentation of results (in italics).

## Results

Overall 39 participants (19 males, 20 females) were interviewed including 14 GP registrars, 12 GP supervisors, and 13 practice managers. All participants were aged from 20 to 50 years. There were 15 Australian Medical Graduates (AMG) and 11 International Medical Graduates (IMG). See Table 2.

**Table 2:**
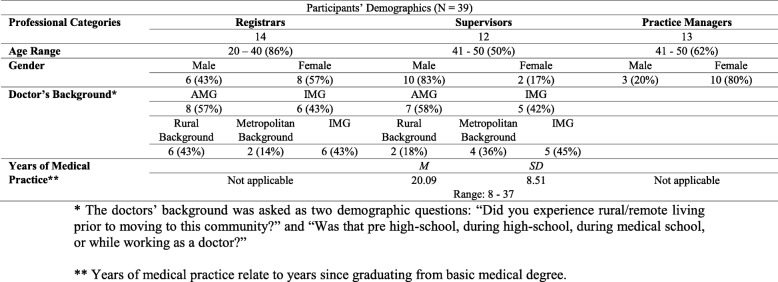
Demographic Profile of Interviewed Participants

Insight from all participants interviewed is presented across four key themes: 1) supervision in underserved communities, 2) impact of working in small, underserved, remote contexts, 3) work-life balance, and 4) fostering sustainable remote practice. (See Table 3).

**Table 3:**
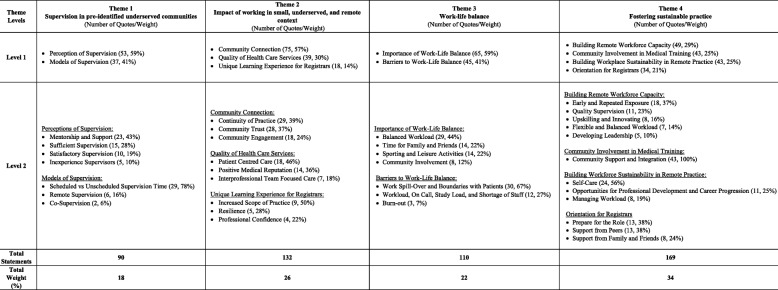
Summary of Themes and Sub-themes

### Theme 1 - supervision in pre-identified underserved communities

Most comments related to perceptions of supervision and emphasised the impact of supervision for providing mentorship and support during registrar training. One registrar commented they chose to train in the location for the quality of supervision available across the community – “It is the reason I came out”. Another registrar noted, however, that supervisors were busy in the remote practice context though they found ways to stay in touch with registrars during busy daily routines, including methods to stay in touch through a “*quick text”*. For trainees needing more support and to develop the resilience for remote practice, supervisors recognised the need to be available “*on the ground”*, and “*on the run*” including the middle of the night for on-call work. Over a quarter of responses raised the issue of sufficient supervision through “*being able to have access to good supervision in the general practice and at the hospital”*. To a lesser extent, some raised issues of remote areas having inexperienced supervisors who are *“not much more experienced than you are - junior people*”. Although most participants discussed the supervision schedule at an operational level, there was no clear consensus about an optimal model of supervision, including the best arrangements for scheduled versus unscheduled “teaching-learning” time outside of delivering the much-needed clinical services in these communities. Additionally, options of using remote supervision and co-supervision models were only raised by a couple of participants, rather than being noted as a possible option for building capacity in different communities. Table 4 exemplifies a range of other comments by various participants.

**Table 4:**
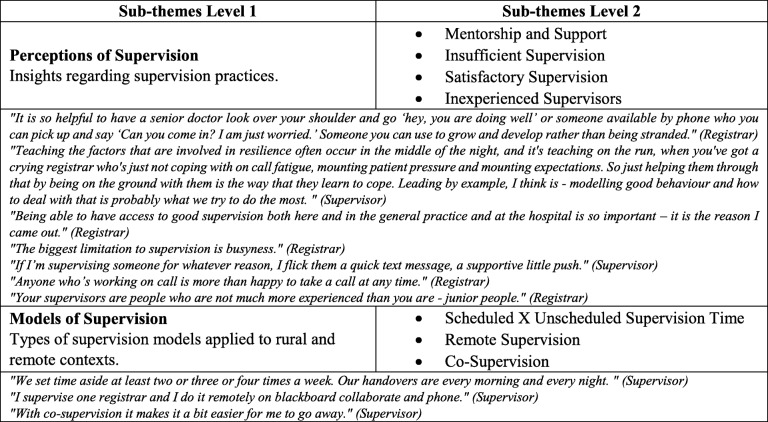
Theme 1 - Supervision in pre-identified underserved communities

### Theme 2 - impact of working in small, underserved, and remote context

The impact of working in small, underserved, and remote communities was discussed in relation to the community, its health service quality, and the unique learning experiences it offered for registrars. The presence of registrars in these communities was perceived by both registrars and supervisors to contribute to health outcomes and continuity of practice to “*make a difference*”. One practice manager also noted the centrality of medical care to the community *“if we didn’t have a doctor, we wouldn’t have a community”*. Community trust and to a lesser extent community engagement emerged as important. Registrars who started a community triathlon club in their community are an example community participation. From the health service perspective, having registrars training in the town facilitated patient-centred care, a positive medical teaching and learning environment, “*quality of service*”, and reputation for good healthcare, improving the perception of the community having skilled doctors, and facilitated inter-professional team-focussed care. The substantial skill demands required were observed by a practice manager who stated that *“some of them* [/registrars/] *come out – they think it’s a small sleepy town…don’t realise the emergency experience that they need”*. Undertaking supervised practice in these communities, registrars were noted to work across an increased scope of practice, develop professional and personal resilience and increase their professional confidence. A registrar noted *“you’ve got real ability to just practice at the top of your level”*. Additionally, a supervisor noted that extensive team work is an important lesson for GP registrars - *“the team is everything”*. Overall, remote underserved communities could focus on engaging registrars and supervisors and promote sustainable work-life practice models for their doctors. Table 5 exemplifies a range of other comments on this theme.

**Table 5:**
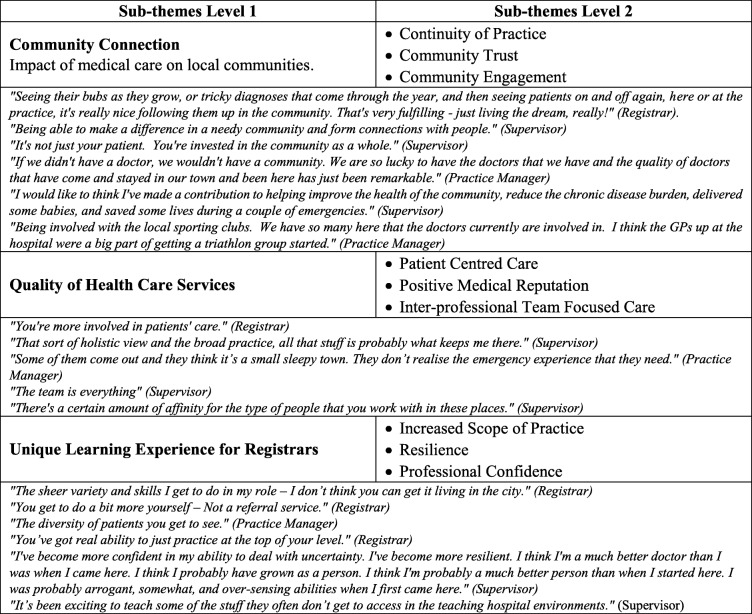
Theme 2 - Impact of Working in small, underserved, and remote context

### Theme 3 - work-life balance

The importance of work life balance in these underserved communities was strongly emphasised. Issues which came up for both supervisors and registrars were trying to balance workload, time for family and friends, sporting and other leisure activities, and community involvement. Supervisors perceived that they were role models of these factors for registrars. Challenges to achieving a work life balance were mainly mentioned by registrars and supervisors and included the problem of work spill-over into time off and boundaries with patients seen in the community, as well as workload, on-call, study load and a shortage of clinical staff. A few participants also mentioned burnout as having the potential to impact their retention and registrars recognised the importance of maintaining and role-modelling the balance of work for sustainability. Some of the key factors driving work life balance in these communities were the amount of quality time with family, doing other activities in the community and workload expectations including on-call requirements. Table 6 exemplifies a range of other comments on this theme.

**Table 6:**
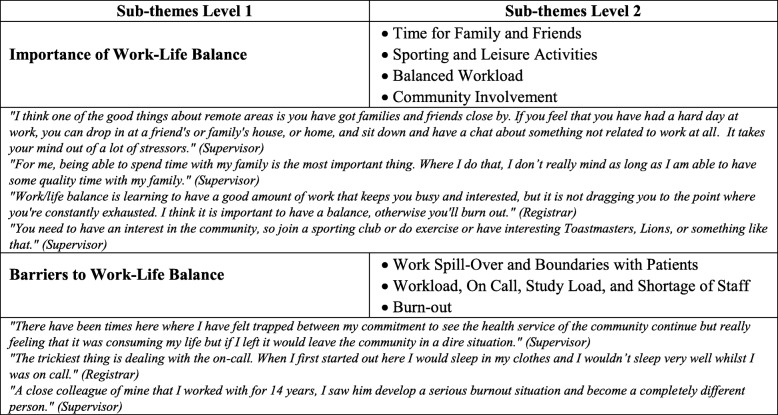
Theme 3 - Work-life balance

### Theme 4 - fostering sustainable practice

Sustainable practice was a strong theme noted by registrars, supervisors, and practice managers impacting the attraction, training, and retention of GPs. Developing the overall local and visiting workforce capacity, engaging community involvement in recruitment and orientation and promoting sustainable workforce practices were all thought to enhance sustainability. Early and repeated exposure of medical students and junior doctors to these communities and fostering the positive aspects of a remote lifestyle were thought to contribute to building a local critical mass and attract more GPs to remote practice. Other ways to potentially increase registrar supply are to foster the quality of supervision in remote medical practice by retaining and supporting supervisors who have remote experience, provide supportive mentors and enhance supervision through co-supervision models. To maintain the current remote GP supervisor workforce, many who were reaching mid-career considered it important to ensure opportunities to upskill and maintain existing skills.

In terms of the learning model in these remote towns, the practice managers specifically suggested building a flexible and balanced workload for both registrars and supervisors to allow for teaching time. Teaching opportunities could be facilitated in the busy environment by more dedicated “educational resource”. The health service leadership in the town was also considered important for supporting sustainable practice by the local GPs, thereby enabling their active engagement in supervision and leadership roles. One registrar explained that it “*makes a big difference in a country hospital having an established administration staff with strong leadership”*. Further, a supervisor explained that *“trying to build leadership and executive structure within our rural medical workforce is something that is needed”.* Having time off and becoming part of the community were also important elements for sustainable practice, and therefore for attracting registrars and retaining supervisors and registrars.

Inherent to registrar learning in the remote setting were developing self-care strategies, enabling opportunities for professional development and career progression relative to personal career interests, and exploring strategies for managing the workload. More registrar orientation to the clinical practice environment was thought to be helpful by a practice manager who explained that “*it would be really beneficial if people came out before and had a look”*. Alternatively, having peer support from other registrars working in a similar role and supervisors engaging with them prior to and throughout their training. It was also important that registrars had mechanisms to receive support and comradery from family, friends, and a broader social network. A supervisor stated *“we have got two guys who have moved there, and the wives are friends and the two boys are great friends from university years. It’s a thumping success”*. The remote underserved communities could focus on engaging registrars and supervisors and promote sustainable work-life practice models for their doctors. Table 7 exemplifies a range of other comments on this theme.

**Table 7:**
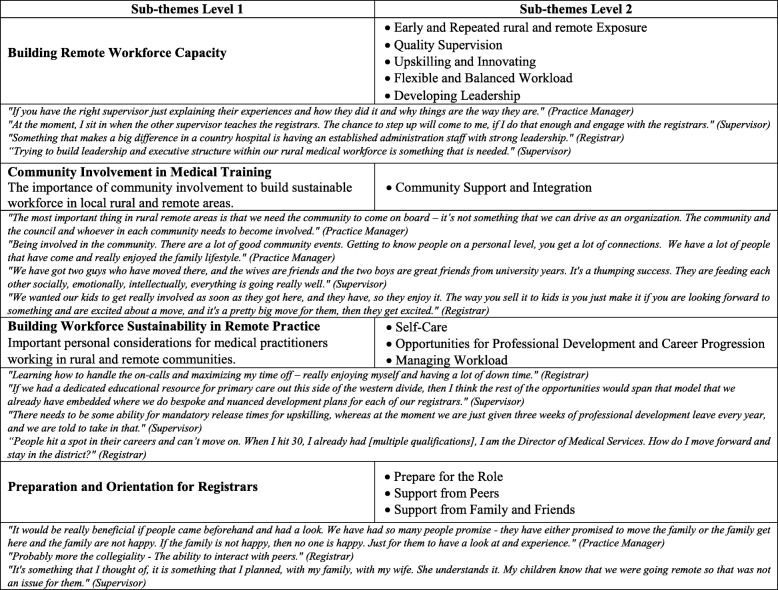
Theme 4 - Fostering Sustainable Practice

## Discussion

This study provides important new insights into the factors that relate to developing GP training capacity in remote areas that need more primary care services for their population’s needs. Four main themes provide guidance as to the direction of investment needed to build general practice workforce and training opportunities in this context. Firstly, supervision is of high quality in this context and supervisors are important role models and mentors, but supervisors are busy working across the community. It was apparent that there was no consistent framework for supervision across the various remote communities. It is important to tailor supervision models to the needs of individual communities including considering options such as remote and co-supervision models, as a means of enabling regular access to supervisors, particularly in this setting where registrars work at the top of their skills and supervisors are time pressured due to clinical demands [26, 27]. To some extent, registrar selection for remote placements may overcome this issue. The registrars in this study were functioning at high levels and knew when to ask for help, however, this may not lead to consistent supervision, depending on the registrar and their willingness to ask for help.

Remote towns provide a unique teaching and learning context for registrars to learn about GP practice through the lens of a community connection seeing the impact of their work on the quality of health care services and on people who really need their services. Providing GP training in remote communities who need more primary care services is good for the community and provides a “richness” of experience for the learners with remote exposure enhancing their scope of practice, feeling of accomplishment, and resilience.

Having a critical mass of doctors was a key issue enabling balanced and flexible work schedules to be modelled by supervisors resulting in attracting and retaining registrars and a sustainable remote supervision model. Critical mass could be developed encouraging groups of medical students and registrars to train together, including early and repeated training opportunities in remote communities as multiple placements throughout medical school, in the early postgraduate years, and during vocational training [22]. Retaining remote supervisors through their careers requires upskilling provisions and innovative work and supervision models to promote career diversification whilst sustaining work in the same remote community [28]. The quality of remote supervision and registrar learning opportunities is potentially a key attractor for medical students and registrars. However, equally important is enhancing this quality by investing in supervisor up-skilling and supporting sustainable working conditions.

Practice managers, in particular, recommended ensuring flexible and balanced workloads for both registrars and supervisors to mitigate the local GP medical workforce shortage. Maintaining doctor’s well-being is an important consideration [29, 30]. Also, it is important that educators and supervisors foster leadership skills in their registrars due to its importance in rural and remote communities [30].

Registrars benefit from the challenge of remote practice and become resilient through optimising opportunities to increase their scope of practice and accelerate their careers [28]. Preparing registrars for their role as rural GPs by engaging with and exploring the community prior to re-locating and having support from other registrars and supervisors prior to and throughout their training is also important as is the support of family and friends.

Encouraging local communities to participate in inducting and integrating registrars and supervisors into the local lifestyle through community inductions, community integration, non-monetary incentives, and cultural training is a critical strategy for success. Overall, the findings from this study, although extracted from interviews in Australian rural and remote communities, may apply to the needs of rural areas worldwide as a way of developing GP training capacity and increasing the workforce in underserved areas.

### Limitations

The scope of the present study is restricted to the communities and stakeholders where the interviews were undertaken. However, these communities were all pre-identified as needing more GP services to address population health needs, and so this paper contributes to the literature on this topic. Towns were sampled in different geographic areas to overcome this potential weakness and the characteristics of these towns is presented. Themes were triangulated by using a range of supervisor-registrar-practice stakeholders until saturation was reached.

## Conclusion

This study provides the first empirical data exploring how supervised general practice training capacity in communities that are underserved may be enhanced. Key factors are: building tailored supervision systems/frameworks across the remote community which include supervisor training, engaging registrars in the unique learning experience of working in these locations, managing work-life balance, and building registrar resilience while ensuring sustainable practice models. This study provides evidence for building capacity in general practice training settings in remote areas which require more primary care services for their population.

## Abbreviations

Australian Medical Graduates (AMG): Doctors who completed basic medical training in an Australian medical school.
Australian Standard Geographical Classification – Remoteness Areas (ASGC-RA): A geographical classification system which defines locations on a scale with respect to their remoteness (physical distance to nearest service centre with 1 classified as Major City, 2 as Inner Regional, 3 as Outer Regional, and 4-5 as Very Remote.
Districts of Workforce Shortage (DWS): Areas classified as having less general practice services than the population average, based on Medicare data.
General Practitioners (GPs): Doctors or physicians who are qualified (fellowed with a specialist medical college following specific post-graduate training) to work in primary care and/or hospitals.
Generalist Medical Training (GMT): A GP training organisation which operates as one of nine decentralised regional organisations across Australia. It provides GP training across over 90% of the large state of Queensland, much of it in rural and remote locations.
Index of Access (IA): A score which delineates level of access to services based on the volume of GP services and population characteristics within floating catchments.
International Medical Graduates (IMG): Doctors completed their basic medical training in another country and now working in Australia.
Modified Monash Model (MMM): A more recent geographical classification system adopted in Australian policy in 2015. This classification defines locations according to their population size and remoteness with 1 being Major City, 2 being >50,000 Population, 3 being 15-50,000 population, 4 being 5-15,000 population, and 6-7 Remote or Very Remote.
Socio-Economic Indexes for Areas (SEIFA): A classification system used nationally as the relative score of socio-economic advantage or disadvantage, based on five-yearly census data.
